# Multimodal Imaging for Total Anomalous Systemic Venous Drainage Diagnosis and Preoperative Planning: A Case Report and Literature Review

**DOI:** 10.3389/fcvm.2022.786278

**Published:** 2022-04-25

**Authors:** Mingyan Ding, Huihui Zhang, Dandan Sun, Qiang Li, Ni Jiao, Fang Zhu

**Affiliations:** Department of Cardiovascular Function, People's Hospital of China Medical University, People's Hospital of Liaoning Province, Shenyang, China

**Keywords:** total anomalous systemic venous drainage, multimodal imaging, echocardiography, coronary sinus atrial septal defect, computed tomography angiography

## Abstract

Total anomalous systemic venous drainage (TASVD) is a rare congenital heart malformation. Here, we report a case of a 40-year-old male patient who had a total anomalous systemic venous drainage. It was diagnosed as the TASVD for the first time through multimodal imaging combined Transthoracic (TTE), transesophageal (TEE) and three-dimensional (3D-TTE) echocardiography, contrast echocardiography and computed tomography angiography (CTA). We review 15 published reports on TASVD and summarize the ultrasonographic characteristics. After intracardiac repair through ectopic venous drainage in cardiac surgery, the patient's cyanosis symptoms were alleviated greatly. Echocardiography was the first-line examination for TASVD. Multimodal imaging combined TTE, TEE, 3D TEE, contrast echocardiography and CTA was necessary for confirmed diagnosis of TASVCD.

## Introduction

Anomalous systemic venous drainage is a rare type of congenital heart malformation and has a 5% incidence rate among all congenital heart diseases (1). It is divided into partial systemic venous return anomalies and total anomalous systemic venous drainage (TASVD). The definition is that the drainage of all systemic veins (SVC, IVC, and the coronary sinus) to the LA. TASVD may also be part of heterotaxy syndrome (2). We report a case of TASVD diagnosed by multimodal imaging and was successfully corrected by surgery.

## Case Report

A 40-year-old male reporting “thirty years chest pain and palpitation after exercise, which got worsened for five days” was admitted to our hospital. Echocardiography revealed atrial septal defect in 2009, but it was untreated. Electrocardiogram revealed (1) sinus bradycardia, (2) high voltage in chest leads, and (3) flat T waves in leads I, III, and aVL. His physical examination revealed stunted growth, cyanosis of lips, 53.4 mmHg partial pressure of oxygen, and 88.6% blood oxygen saturation level. A loud, soft pan systolic murmur could be heard over the cardiac apex. His past medical history included cerebral infarction and migraine. 1 month before hospitalization, the patient had acute cerebellar infarction that improved after medical treatment. Biochemical parameters were within normal limits.

**Figure 1:**
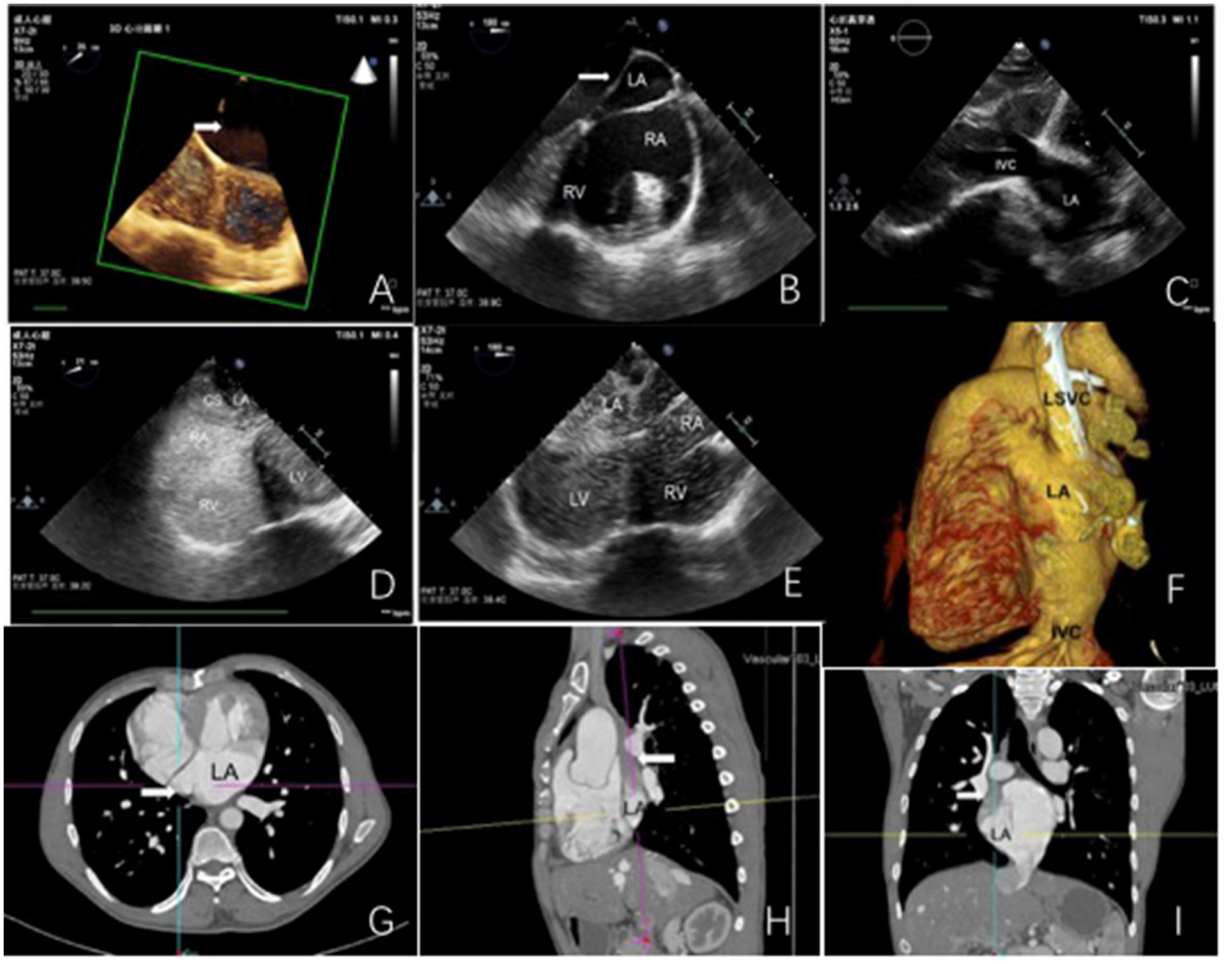
Echocardiography and computed tomography angiography. **(A)** complete coronary sinus atrial septal defect (white arrowheads). **(B)** white arrowheads show an abnormal conduit. **(C)** A connection between the inferior vena cava and the left atrium. **(D)** saline microbubbles injected into the lower limb vein expressed first in the LA and left ventricle (LV), and then in the RA and RV. **(E)** saline microbubbles injected into the right elbow vein expressed almost simultaneously in the LA and RA. **(F)** CT shows the left superior vena cava and the inferior vena cava draining into the LA. **(G–I)** CT shows the superior vena cava (white arrowheads) draining into the.

Transthoracic (TTE), transesophageal (TEE) and three-dimensional (3D-TTE) echocardiography revealed (1) coronary sinus dilatation and a complete coronary sinus atrial septal defect (ASD) (Figure 1A, Supplementary Videos 1, 2), (2) an anomalous conduit enters left atrium (LA) at its roof (Figure 1B), and (3) the inferior vena cava (IVC) opens into LA (Figure 1C) (4) the superior vena cava (SVC) is not connected with the right atrium (RA). Contrast echocardiography revealed (1) saline microbubbles injected to the left elbow vein expressed first in the LA and then in the RA and right ventricle (RV) (Supplementary Video 3); (2) saline microbubbles injected into the right elbow vein expressed almost simultaneously in the LA and RA (Figure 1D, Supplementary Video 4); (3) saline microbubbles injected into the lower limb vein expressed first in the LA and left ventricle (LV) and then in the RA and RV (Figure 1E, Supplementary Videos 5, 6). Arch was normal. Accordingly, these were diagnosed as complete coronary sinus ASD, persistent ectopic venous draining from the left SVC to the LA, ectopic venous draining from the IVC to the LA, and ectopic venous draining from the right SVC to the LA. Hepatic veins were draining into the IVC, and pulmonary veins were draining into the LA. ECG-gated computed tomography angiography (CTA) demonstrated complete coronary sinus ASD, persistent left SVC draining into the LA (Figure 1F), right SVC draining into the LA (Figures 1G–I), and IVC draining into the LA (Figure 1F). A contrast medium was injected in the left elbow vein. Finally, it was diagnosed as total anomalous systemic venous drainage (TASVD) through cardiac surgery, and was consistent with the multimodal imaging (TTE, TEE, 3D TEE, contrast echocardiography and CTA).

During the cardiac surgery, we saw a complete coronary sinus atrial septal defect, the right superior vena cava being slightly thinner and draining into the coronary sinus opening in the right atrium, persistent left superior vena cava draining into the left atrium, which opened between the left atrial appendage and the left superior pulmonary vein, and the inferior vena cava opening between the mitral valve and the right inferior pulmonary vein. Both atrial appendages were normal in morphology. Thus, we performed a repair of the atrial septal defect and an intracardiac repair through ectopic venous drainage.

After the operation, the patient recovered well: his face and lip color was normal; his partial pressure of oxygen was 97.7 mmHg, and his blood oxygen saturation level was 97.8%. 3 months after the operation, TTE revealed that his cardiac size returned to normal. At 1-year follow-up, the man was doing well and had normal oxygen saturation and unobstructed systemic venous drainage.

## Discussion

TASVD is a rare type of congenital heart malformation. TASVD drainage requires the presence of a left-to-right shunt (PDA, ASD, or VSD) to allow the systemic venous return to reach the pulmonary circulation (3). Functional drainage of systemic venous blood into the left atrium across an atrial septal defect has been described. The mechanisms is that TASVD probably results from the sinus venosus being incorporated into the LA (4) or that the right valve of the systemic venous sinus fails to regress (5). There are three subsets of TASVD based on the type of vena cava cannulation (2). In type I, the IVC is not interrupted and conventional cardiopulmonary bypass can be performed. In type II, the IVC is interrupted, and single cannulation of the SVC would suffice for venous drainage. In type III, the IVC drains to an accessory chamber like coronary sinus. According to the above classifications, our case belongs to type I. Electronic databases including Pubmed, Web of Science, and Medline were searched to identify TASVD, from inception to May 2021. We reviewed 13 published reports on TASVC and summarized the ultrasonographic characteristics in Table 1.

**Table 1:** Ultrasonographic characteristics of the 15 cases of total anomalous systemic venous drainage.

**Authors**	**Year**	**Age**	**Sex**	**Symptom**	**RSVC**	**Persisitent LSVC**	**IVC**	**Isomerism**	**ASD**	**VSD**	**Arch**	**PDA**
Samaan (6)	2016	20y	M	shortness of breath,cyanosis	LA	LA	LA	Not committed	YES	-	Normal	-
Agarwal (7)	2014	12y	M	Asymptomatic	LA	-	interrupted	-	-	-	Normal	-
Simha (8)	2012	11y	F	cyanosis, fatigue, palpitations	LA	-	interrupted	left atrial isomerism	YES	-	Normal	-
Khandenahally (9)	2013	11y	F	fatigue, cyanosis	LA	-	interrupted	-	YES	-	Normal	-
Kao (10)	1983	4y	F	Cyanosis	LA	-	interrupted	-	YES	YES	Normal	-
Awasthy (2)	2014	5y	F	Cyanosis	LA	-	LA	left atrial isomerism	YES	YES	Right arch	-
		5y	F	Cyanosis	LA	-	interrupted	left atrial isomerism	YES	YES	Right arch	-
		2y	M	Cyanosis	LA	LA	interrupted	left atrial isomerism	YES	-	Normal	-
Yildirim (11)	2014	2d	M	Cyanosis	-	LA	interrupted	left atrial isomerism	YES	YES	Normal	-
Roberts (4)	1972	3y	F	Cyanosis	LA	LA	interrupted	-	YES	-	Normal	-
Vo (12)	2015	7y	F	dyspnea, cyanosis	LA	LA	interrupted	left atrial isomerism	-	YES	Normal	-
Vaidyanathan (13)	2016	9y	F	Dyspnea	LA	-	LA	-	YES	-	Normal	-
Zhang (14)	2009	33m	F	Cyanosis	LA	LA	LA	-	YES	YES	Normal	-
Devendran (15)	2013	27y	F	Cyanosis	LA	-	LA	-	YES	-	Normal	-
Lazzarin (16)	2009	1d	F	mild dyspnea, cyanosis	LA	-	LA	-	YES	-	Normal	YES

*M, male; F, female; LA, left atrium; RSVC, right superior vena cava; IVC, inferior vena cava; ASD, atrial septal defect; VSD, ventricular septal defect; PDA, patent ductus arteriosus*.

Because of individual differences and lack of specific laboratory tests, medical diagnosis of TASVD is usually challenging. Up to now, echocardiography is still the most common clinical examination. In this case, multimodal imaging was performed to diagnose TASVD. Echocardiography manifestations are as follows: (1) two-dimensional echocardiography shows that the LA is significantly enlarged and that the right heart system is relatively small; (2) communication between the SVC and the LA was not seen in the conventional ultrasound view of two-dimensional transthoracic echocardiography; (3) two-dimensional transesophageal echocardiography clearly shows that the SVC and the IVC are not communicating with the RA; (4) right heart contrast echocardiography can help assess the connection between veins and atriums effectively, and the communication between the SVC, the IVC, and the LA; (5) three-dimensional transesophageal echocardiography can directly display the communication between the abnormal duct and the LA. TTE, TEE, and 3D TEE are considered useful approaches to help observe the hind and inside of heart structures. Contrast echocardiography can help assess the connection between veins and atriums effectively. CTA examination is an invasive and reliable way to examine congenital heart diseases and can provide more comprehensive and three-dimensional cardiovascular imaging. Through CTA examination, the connection between pericardia great vessel system and heart chambers can be assessed. The main advantage of CT is its high-resolution tridimensionality, and a 3D image could add an interesting view of the cardiac anomaly. This case was finally diagnosed and compressively expressed by multimodal imaging (TTE, TEE, 3D TEE, contrast echocardiography, and CTA). Noninvasive examinations such as MRI can be used to assess defects of cardiovascular walls or help observe flow signals from vein to the LA without using contrast media, especially in pediatric populations with congenital heart disease because of lack of ionizing radiation (17).

TASVD is a rare and usually hard to detect congenital heart malformation. As the first diagnostic examination, ultrasound examination has its unique advantages in TASVD diagnosis. In the face of cases of complex anomalous cardiac venous system connection, we need to perform multimodal imaging to confirm the diagnosis of TASVD.

## Data Availability Statement

The original contributions presented in the study are included in the article/Supplementary Material, further inquiries can be directed to the corresponding author/s.

## Ethics Statement

Written informed consent was obtained from the individual(s) for the publication of any potentially identifiable images or data included in this article.

## Author Contributions

MD, HZ, NJ, and DS: study concept, acquisition of data and figures, and writing of the manuscript. QL and FZ: study concept and critical revision of the manuscript for intellectual content. All the authors cared for the patient and contributed to the writing of the report.

## Funding

This study was supported by the Liaoning Province Xingliao Talents Plan Project (XLYC2005007) and Science and Technology Fund of Liaoning Province (20180530109).

## Conflict of Interest

The authors declare that the research was conducted in the absence of any commercial or financial relationships that could be construed as a potential conflict of interest.

## Publisher's Note

All claims expressed in this article are solely those of the authors and do not necessarily represent those of their affiliated organizations, or those of the publisher, the editors and the reviewers. Any product that may be evaluated in this article, or claim that may be made by its manufacturer, is not guaranteed or endorsed by the publisher.

## Supplementary Material

Supplementary Videos 1, 2 Coronary sinus dilatation and complete coronary sinus atrial septal defect (ASD).

Supplementary Video 3 Saline microbubbles injected to left elbow vein expressed first in LA, and then in RA and right ventricle.

Supplementary Video 4 Saline microbubbles injected into right elbow vein expressed almost simultaneously in LA and RA.

Supplementary Videos 5, 6 Saline microbubbles injected into lower limb vein expressed first in LA and left ventricle (LV), and then in RA and RV.

## Abbreviations

LA: left atrium
IVC: inferior vena cava
RA: right atrium
RV: right ventricle
LV: left ventricle
CS: coronary sinus
LSVC: left superior vena cava.
